# Psychopathy traits and their link to emotion recognition impairments in conduct disorder

**DOI:** 10.1002/jcv2.70055

**Published:** 2025-09-30

**Authors:** Gregor Kohls, Anka Bernhard, Graeme Fairchild, Christina Stadler, Lucres Jansen, Stephane A. De Brito, Veit Roessner, Christine M. Freitag, Kerstin Konrad, Erik M. Elster

**Affiliations:** ^1^ Department of Child and Adolescent Psychiatry Faculty of Medicine TUD Dresden University of Technology German Center for Child and Adolescent Health (DZKJ) partner site Leipzig/Dresden Dresden Germany; ^2^ Department of Psychology University of Bath Bath UK; ^3^ Department of Child and Adolescent Psychiatry Psychiatric University Hospital University of Basel Basel Switzerland; ^4^ Department of Child and Adolescent Psychiatry VU University Medical Center Amsterdam the Netherlands; ^5^ Centre for Human Brain Health School of Psychology University of Birmingham Birmingham UK; ^6^ Department of Child and Adolescent Psychiatry, Psychosomatics and Psychotherapy University Hospital Frankfurt Goethe University Frankfurt am Main Germany; ^7^ Child Neuropsychology Section Department of Child and Adolescent Psychiatry, Psychosomatics and Psychotherapy RWTH Aachen University Aachen Germany; ^8^ RWTH Aachen & Research Centre Juelich JARA‐Brain Institute II, Molecular Neuroscience and Neuroimaging Juelich Germany

**Keywords:** callous‐unemotional traits, conduct disorder, emotion recognition, grandiose‐manipulative traits, impulsive‐irresponsible traits, psychopathy

## Abstract

**Background:**

Neurocognitive models suggest that callous‐unemotional (CU) traits in youths with conduct disorder (CD) are linked to emotion recognition impairments, particularly in identifying distress emotions like fear and sadness. However, CD may be accompanied by grandiose‐manipulative (GM) and/or impulsive‐irresponsible (II) traits in addition to CU traits, consistent with the notion that psychopathy is a multifaceted construct. It remains unclear whether subgroups of CD youths with GM or II traits, as well as a combination of multiple psychopathic traits, show distinct patterns of emotion recognition impairment.

**Methods:**

We therefore assessed emotion recognition accuracy, focusing on the six basic emotions, in 538 youths with CD (315 girls, 9–18 years) who were assigned to one of eight mutually exclusive subgroups based on high or low scores (above/below the 75th percentile cut‐offs) on CU, GM, and II traits as assessed by the Youth Psychopathy Inventory (YPI) self‐report.

**Results:**

Support vector machine analyses supported the validity of the subgroup assignment based on YPI cut‐off scores (sensitivities/specificities ≥75%–100%). Multinomial logistic regression models revealed that the CD subgroup with high levels across all three psychopathy traits had the most pervasive impairment in terms of higher error rates for recognizing sadness, disgust, and surprise. Notably, high CU traits were not consistently associated with impairments in fear and sadness recognition. Instead, CD youths with both high GM and II traits, but normal CU traits, showed impaired fear recognition.

**Conclusion:**

These findings challenge existing models that prioritize CU traits as the main driver of emotion recognition impairments in CD. Instead, different psychopathy traits appear to contribute distinctively to such impairments, including the recognition of distress emotions. This suggests that CU traits alone may not sufficiently explain the neurocognitive heterogeneity in emotion recognition seen in youths with CD.

## Key Points

**What's known**

- Emotion recognition impairments, especially for distress emotions like fear and sadness, are commonly linked to callous‐unemotional (CU) traits in youths with conduct disorder (CD).
- Psychopathy is multifaceted, encompassing not only CU traits but also grandiose‐manipulative (GM) and impulsive‐irresponsible (II) traits that can accompany CD.

**What's new**

- In a large sample of girls and boys with CD, distinct patterns of emotion recognition impairment emerged across psychopathy trait profiles.
- Youths high across all three traits showed the most pervasive impairments, while those high in GM and II traits (but not CU) were specifically impaired in fear recognition. CU traits were not consistently linked to impairments in recognizing distress emotions.

**What's relevant**

- CU traits alone do not account for emotion recognition deficits in CD, highlighting the need for more nuanced, trait‐specific neurocognitive models.

## INTRODUCTION

Conduct disorder (CD) is one of the most prevalent mental disorders in youths. It is characterized by recurrent severe antisocial and aggressive behaviors that violate age‐appropriate social norms and others' rights (American Psychiatric Association, 2013). There is an urgent need for clinical approaches and tools that better capture the vast phenotypic heterogeneity of the disorder and to identify subtypes with different risk factors, developmental trajectories, etiologies, and treatment needs (Fairchild et al., 2019). Current diagnostic approaches focus heavily on the age‐of‐onset and severity of CD symptoms as well as the presence or absence of CU traits (i.e., reduced guilt and empathy, shallow emotions, and lack of concern regarding one's own performance), as one of the most commonly studied facets of psychopathy. Other potentially relevant individual differences in interpersonal or behavioral traits that often accompany CD have been largely neglected in the relevant clinical research (Salekin, 2016b). As a result, important aspects of the disorder's phenotype beyond age‐of‐onset, severity, and CU traits may be overlooked, leading to incomplete diagnoses and thus less effective treatment strategies.

Of particular clinical importance here is “psychopathy,” which has emerged as a promising construct for more precisely describing the phenotype of those diagnosed with CD (Frick, 2009; Salekin, 2016a; but see also the controversy regarding the use of this construct for youths, as discussed by Frick, 2022; Salekin, 2022). Various studies have consistently identified three distinct but interrelated psychopathy traits in youths, in addition to CD symptoms, which parallel those identified in adults with psychopathy: CU traits, grandiose‐manipulative (GM) traits, and impulsive‐irresponsible (II) traits. Although all three of these traits have been associated with core or accompanying symptoms of CD (e.g., aggression, property destruction, bullying, or substance use) (Fanti et al., 2018; Frick et al., 2000), there is a lack of research on utilizing these traits for subtyping youths with this disorder. This gap may prevent a more comprehensive and refined understanding of the disorder's broad phenotype.

Although psychopathy is a multifaceted construct that includes multiple traits, it is unfortunate that research on psychopathy subtypes in youths with CD has focused primarily on CU traits (Fanti et al., 2018). As a result, of the three facets of psychopathy, only CU traits were incorporated in the DSM‐5 and ICD‐11 classification manuals in the form of the “with Limited Prosocial Emotions” (LPE) specifier/subtype. Research has shown that qualifying for the LPE subtype of CD is associated with an early onset and particularly severe and chronic CD symptoms, which requires specific treatment planning (American Psychiatric Association, 2013).

However, in the context of the introduction of the LPE specifier in DSM‐5 and ICD‐11, the central importance of CU traits in subtyping CD has been repeatedly questioned. It has been suggested instead that CD might be better specified by considering all three psychopathy traits—not only CU traits but also GM traits (i.e., a sense of superiority, self‐importance, and a tendency to dominate and manipulate others) and II traits (i.e., a tendency towards reckless sensation seeking, the need for stimulation, and the avoidance of monotony and boredom) (Salekin, 2016a). This recommendation has been made despite the fact that GM and II traits have received significantly less attention in the research literature compared to CU traits. Notably, though, similar to CU traits, both GM and II traits can be measured reliably even in young children (e.g., preschoolers), remain relatively stable throughout development, and appear to be closely related to severe manifestations of CD symptoms (López‐Romero et al., 2022; Salekin, 2016a).

According to Salekin (2016a, 2016b, 2017), this research gap can be partly attributed to the general yet unproven assumption that psychopathy in youths with CD has only one single core phenotype—namely high CU traits (e.g., Blair, 2013; Byrd et al., 2014). The existing literature suggests otherwise: Both cross‐sectional and longitudinal studies show that the combination of high levels of CU, GM, and II traits is a much stronger predictor of severe and stable CD symptoms than each of the three traits alone (reviewed in Fanti et al., 2018; Salekin, 2016a, 2016b, 2017); a finding that has been replicated in youths of both sexes, including community, clinic‐referred, and forensic samples. In fact, the vast majority of youths with (or at risk for) a clinical diagnosis of CD appear to be characterized by a combination of high CU, GM, and II traits, and high CU traits alone do not appear to sufficiently account for heterogeneity within CD, including disorder severity (Fanti et al., 2018; Frick et al., 2000; López‐Romero et al., 2022; Ribeiro da Silva et al., 2019). As a result, some researchers have advocated for incorporating all three psychopathy traits into future revisions of diagnostic and classification systems (i.e., DSM and ICD). This approach could refine the diagnostic category of CD by describing and subtyping it on the basis of a broader array of psychopathy traits, potentially paving the way for the introduction of new and/or revised specifiers (Salekin, 2024). Others argue that this would be premature but call, at the very least, for more systematic research on the multiple psychopathy traits in youths with CD (Frick, 2022).

Influential neurocognitive models suggest that emotion recognition impairments (i.e., difficulties in identifying facial expressions, such as the core distress emotions of fear and sadness, due to aberrant amygdala‐ventromedial prefrontal cortex responses) is a key functional impairment closely linked to the emergence of psychopathy in youths (Blair, 2013)—with these models focusing specifically on CU traits rather than on GM or II traits. Therefore, it is currently unknown whether previous findings of emotion recognition impairments in youths with primarily high CU traits can be generalized to high GM traits and/or high II traits of psychopathy, the extent to which specific emotion recognition impairments may be associated with certain psychopathy traits, or whether CD youths with high levels across all three psychopathy traits will show the most extensive emotion recognition impairments (Díaz‐Vázquez et al., 2024).

The current study addresses this research gap by reanalyzing neurocognitive task performance data on facial emotion recognition from the FemNAT‐CD cohort in relation to the multiple facets of psychopathy in youths with CD (i.e., CU, GM, II traits). Based on previous work (Dawel et al., 2012; Díaz‐Vázquez et al., 2024), we hypothesized that high CU traits in youths with CD would be associated with impairments in general emotion recognition, but most pronounced for distress emotions, such as fear and sadness—although there are also some conflicting results in this regard (reviewed in Díaz‐Vázquez et al., 2024). We further expected that the combination of high levels across all three psychopathy traits may be associated with the most pervasive (e.g., widespread) emotion recognition impairments. However, some of these effects may only be observed for more difficult‐to‐recognize expressions (i.e., less intense, more subtle presentations of an emotion), as a few studies suggest that youths with psychopathy require a “clearer” emotional stimulus to achieve accurate identification (Bowen et al., 2014; Blair et al., 2001); although existing data are too mixed to strongly support such a prediction (Díaz‐Vázquez et al., 2024). Furthermore, no specific hypotheses about high GM and II traits, as well as possible additive effects of two psychopathy traits, and their associations with emotion recognition impairments can be derived from the available literature (Díaz‐Vázquez et al., 2024; Levantini et al., 2023).

To systematically investigate how specific constellations of psychopathy traits relate to emotion recognition impairments, we created eight mutually exclusive subgroups based on 75th percentile cut‐off scores from the CU, GM, and II subscales of the Youth Psychopathy Inventory (YPI). This approach, consistent with previous work (e.g., Colins et al., 2024), enables a clinically motivated and meaningful examination of neurocognitive heterogeneity that goes beyond a single trait or specifier dimension—such as the LPE specifier in DSM‐5.

## MATERIAL AND METHODS

### Participants

This study included 538 youths with CD (315 girls), aged 9–18 years, from the European FemNAT‐CD project (Freitag et al., 2018). Exclusion criteria included intelligence quotient (IQ) < 70, autism spectrum disorders, schizophrenia, bipolar disorders or mania, neurological disorders, and genetic syndromes. All youths fulfilled diagnostic criteria for current CD according to DSM‐IV‐TR criteria (American Psychiatric Association, 2000). Psychiatric diagnoses were determined by trained staff using the Kiddie‐Schedule for Affective Disorders and Schizophrenia–Present and Lifetime version (K‐SADS‐PL; Kaufman et al., 1997), a semi‐structured clinical interview administered separately to participants and their caregivers. Full‐scale IQ was estimated with the Wechsler Intelligence Scales (Wechsler, 1999, 2003, 2008). Socioeconomic status (SES) was estimated based on the International Standard Classification of Education framework, normalized for the participant's country (Pauli et al., 2021). See Kohls, Baumann, et al. (2020) and Kohls, Fairchild, et al. (2020) for psychometric information on these measures.

We used the YPI to capture the three psychopathy traits (Andershed et al., 2002). The YPI is a 50‐item self‐report measure in which each item is answered on a 4‐point Likert scale ranging from “does not apply at all” (1) to “applies very well” (4). Higher scores indicate higher levels of psychopathy. The YPI was used because CU, GM, and II traits are framed as characteristics that appear neutral or even appealing to youths with these traits, rather than framing the items as deficits. The YPI consists of 10 subscales corresponding to the three psychopathy traits (i.e., CU traits scale: callousness, unemotionality, and remorselessness; GM traits scale: dishonest charm, grandiosity, lying, and manipulation; II traits scale: impulsiveness, thrill seeking, and irresponsibility). The YPI and its CU, GM, and II traits scales have shown good psychometric properties in terms of reliability, stability, and concurrent as well as criterion validity and predictive validity (Andershed et al., 2002; van Baardewijk et al., 2008). Cronbach's *α* for the three psychopathy scales of the YPI in the present sample was 0.75 for CU traits, 0.84 for GM traits, and 0.79 for II traits, which are acceptable.

For each psychopathy scale (CU, GM, and II traits), youths with CD were classified into higher‐ or lower‐scoring subgroups based on the 75th percentile cut‐off scores (Fisher et al., 2020): CU = 38, GM = 47, and II = 46. Following Colins et al. (2024), we then identified eight mutually exclusive psychopathy subgroups: CD‐only (below cut‐off on all traits), CD + CU (above on CU only), CD + GM (above on GM only), CD + II (above on II only), CD + CU + GM (above on CU and GM), CD + CU + II (above on CU and II), CD + GM + II (above on GM and II), and CD + CU + GM + II (above on all traits). Table 1 summarizes the groups' main demographic and clinical characteristics. Note that overall, the subgroups did not differ substantially in their demographic and clinical phenotypes. Also note that we do not intend to claim that the subgroups represent diagnostic entities, but they rather serve as theoretical profiles to capture additive and interactive constellations of psychopathy traits. Local ethics committees at each site approved the study protocol. Written informed consent was obtained for all participants.

**TABLE 1:** Main demographic information and clinical characteristics per psychopathy subgroup (overall *N* = 538).

	CD‐only (*n* = 281, 52.2%)	CD + CU (*n* = 45, 8.4%)	CD + GM (*n* = 42, 7.8%)	CD + II (*n* = 49, 9.1%)	CD + CU + GM (*n* = 26, 4.8%)	CD + CU + II (*n* = 18, 3.3%)	CD + GM + II (*n* = 27, 5.0%)	CD + CU + GM + II (*n* = 50, 9.3%)	Group effect *p* value, ES
Girls (%)	57.3^a^	40.0^a^	61.9^a,b^	81.6^b^	61.5^a,b^	55.6^a,b^	74.1^a,b^	48.0^a^	0.002, Cramer's *V* = 0.21
Age (years) M(SD)	14.1 (2.3)^a^	14.4 (2.3)^a,b^	14.3 (2.2)^a,b^	15.3 (1.8)^b^	14.5 (2.4)^a,b^	14.3 (2.2)^a,b^	15.1 (2.7)^a,b^	14.8 (2.0)^a,b^	0.025, Ω^2^ = 0.038
Estimated total IQ M(SD)	94.7 (12.0)	93.0 (12.0)	97.8 (13.6)	94.0 (10.6)	97.7 (14.4)	95.4 (14.1)	94.8 (16.4)	93.9 (11.3)	ns, Ω^2^ = 0.005
Socioeconomic status M(SD)	−0.32 (0.92)	−0.15 (0.67)	−0.05 (0.67)	−0.48 (1.13)	−0.23 (0.86)	−0.64 (0.99)	−0.03 (0.75)	−0.39 (1.36)	ns, Ω^2^ = 0.021
Current CD symptoms M(SD)	5.0 (2.1)^a^	5.6 (2.4)^a,b^	5.3 (2.6)^a,b^	6.1 (2.3)^a,b^	5.7 (2.4)^a,b^	6.4 (2.2)^a,b^	6.4 (2.8)^b^	6.3 (2.7)^b^	<0.001, Ω^2^ = 0.044
YPI M(SD):									
CU traits	28.4 (4.7)^a^	42.3 (4.3)^b^	32.0 (4.1)^c^	27.9 (5.6)^a^	41.7 (3.6)^b^	45.1 (6.2)^b^	32.8 (3.1)^c^	43.0 (3.9)^b^	<0.001, Ω^2^ = 0.68
GM traits	31.7 (7.2)^a^	36.1 (6.1)^b^	51.5 (4.5)^c^	36.2 (6.3)^b^	53.9 (6.1)^c^	38.5 (7.4)^b^	52.7 (5.6)^c^	55.9 (5.9)^c^	<0.001, Ω^2^ = 0.69
II traits	34.4 (6.8)^a^	39.4 (5.2)^b^	38.6 (4.1)^b^	48.9 (2.9)^c^	40.9 (3.0)^b^	49.1 (2.8)^c^	50.1 (3.9)^c^	50.2 (3.2)^c^	<0.001, Ω^2^ = 0.61
Current comorbidities (%):									
ODD	74.0	68.9	81.0	83.7	92.3	94.4	77.8	86.0	ns, Cramer's *V* = 0.16
ADHD	37.0	28.9	38.1	40.8	30.8	66.7	25.9	40.0	ns, Cramer's *V* = 0.14
SUD	12.1^a^	11.1^a,b^	16.7^a,b^	36.7^b^	11.5^a,b^	27.8^a,b^	33.3^a,b^	26.0^a,b^	<0.001, Cramer's *V* = 0.23
MDD	17.4	22.2	9.5	18.4	11.5	44.4	22.2	14.0	ns, Cramer's *V* = 0.16
PTSD	6.8^a^	2.2^a^	4.8^a,b^	4.1^a,b^	11.5^a,b^	27.8^b^	18.5^a,b^	4.0^a,b^	0.004, Cramer's *V* = 0.19
GAD	13.9^a,b^	13.3^a,b^	14.3^a,b^	10.2^a,b^	11.5^a,b^	33.3^b^	11.1^a,b^	0.0^a^	0.035, Cramer's *V* = 0.17

*Note*: Diagnoses were based on the Schedule for Affective Disorders and Schizophrenia for School‐Age Children–Present and Lifetime version (K‐SADS‐PL). CU, GM, and II traits were assessed with the Youth Psychopathic Traits Inventory (YPI). Note though that we only report post hoc results if there were in fact significant between‐group main effects. Information on race and/or ethnicity was not collected in accordance with governmental and ethical guidelines in Germany. It should also be noted that we did not assess the gender identity of the participants, but assigned them to the girls or boys sample based on their sex at birth. *p* values are based on *F* tests or *Χ* ^2^ tests. Cramer's *V*: 0.1 = weak; 0.3 = moderate; 0.5 = strong. Ω^2^: 0.01 = weak; 0.06 = moderate; 0.14 = strong. Subgroups with different superscript indices differed significantly in post‐hoc comparisons (*p* ≤ 0.05, Bonferroni‐corrected, i.e., *p* ≤ 0.05/28 ≤ 0.002), those with the same index did not.

Abbreviations: ADHD, attention deficit hyperactivity disorder; CD, conduct disorder; CU, callous‐unemotional; ES, effect size; GAD, generalized anxiety disorder; GM, grandiose‐manipulative; II, impulsive‐irresponsible; IQ, estimated intelligence quotient; MDD, major depressive disorder; ODD, oppositional defiant disorder; PTSD, post‐traumatic stress disorder; SES, socioeconomic status; SUD, substance use disorder.

### Emotion recognition task

We used the *Emotion Hexagon task* to assess emotion recognition accuracy, including the six basic facial expressions—happiness, surprise, fear, sadness, disgust, and anger (Calder, 1996). We chose this task based on influential models of emotion recognition impairment in psychopathy/CU traits, and because it is widely used in neurocognitive research on emotion processing in developmental psychopathology, including CD (Blair et al., 2014). In addition, the available psychometric information supports both the validity and reliability of the task (Kohls, Fairchild, et al., 2020). For example, the extracted performance variables had acceptable to good internal consistency (Cronbach's *α* ≥ 0.70).

Participants were presented with facial expressions that represented a blend of two emotions. They were then asked to select by mouse click one of six presented labels (i.e., one label per emotion, such as “happy” for the emotion happiness) that best described the emotional content of the presented facial expression. The blends were generated from six emotion pairings arranged in a hexagon (see Pauli et al., 2023): happiness‐surprise, surprise‐fear, fear‐sadness, sadness‐disgust, disgust‐anger, and anger‐happiness. Each blend contained two prototypical expressions in a ratio of 90:10, 70:30, 50:50, 30:70, or 10:90 (e.g., the 90:10 happiness‐surprise blend consisted of 90% happiness and 10% surprise). The “dominant” emotion within a blend was considered the correct response. 50:50 blends were excluded from analysis because neither emotion could be definitively classified as correct or incorrect. Blended expressions were presented individually and in random order on a computer screen for a maximum of 3 s. Participants could take as long as needed to respond and received no feedback about their performance accuracy. Participants completed one practice block, followed by five blocks that each displayed all 30 blended expressions once (6 pairs × 5 blends).

For each participant, we calculated the error rate (i.e., incorrect recognition expressed as a percentage) per emotion at its high intensity (i.e., its 90:10 ratio, or 90% presentations) and its low intensity (70:30, or 70% presentations), hypothetically reflecting two levels of difficulty in recognizing a particular expression (i.e., high‐intensity presentations were considered easier to recognize, and low‐intensity presentations were considered harder to recognize). We consider fear and sadness (and probably disgust) to be “distress” emotions, in line with their theoretical role in neurocognitive models of psychopathy/CU traits in CD.

### Statistical analyses

All statistical analyses were conducted using R (Version 4.3.2). We first applied a confirmatory machine learning (ML) approach, followed by multinomial logistic regressions (MLR) models to investigate the different psychopathy subgroups and their relationship to emotion recognition impairments. In addition, we computed (partial) correlations to explore the dimensional association among the three psychopathy traits, as well as their relation with the emotion recognition error rates for each basic emotion (see Supporting Information S1).

#### Support vector machine

To test whether the predefined psychopathy subgroups could be confirmed through a supervised ML approach based on the participants' continuous YPI trait scores, we applied a support vector machine (SVM). Only the three *z*‐standardized trait scores (CU, GM, II traits) were used as input features. No emotion recognition or other phenotypic variables were included as features to avoid circularity.

The SVM was implemented as a multiclass classification model to serve as a quality control procedure, assessing whether the subgroups defined by 75th percentile cut‐offs could be distinguished based on participants' original continuous YPI trait scores. This confirmatory step was not intended for predictive modeling, but rather to evaluate the internal consistency and validity of the subgrouping strategy applied in the subsequent analyses.

SVM is a widely used ML algorithm designed for classification and regression tasks, particularly effective in handling high‐dimensional data and complex classification problems. The method works by identifying a hyperplane that maximizes the distance between data points from distinct classes in an n‐dimensional feature space (Noble, 2006).

The dataset was split into a training and testing set using an 80:20 stratified sampling ratio to ensure preserved subgroup representation in both sets. Given that YPI scores are continuous predictors, we opted for a linear kernel, as it provides an interpretable decision boundary and facilitates the direct assessment of feature contributions to classification. To ensure robust model evaluation, we implemented 10‐fold cross‐validation. This approach partitions the training data into 10 subsets, iteratively training the model on nine subsets and validating it on the remaining subset. The final model was then applied to the held‐out test dataset to evaluate generalizability.

The classification accuracy of the SVM was assessed using confusion matrices, which provided detailed performance metrics, including sensitivity, specificity, positive predictive value, negative predictive value, and balanced accuracy for each of the eight psychopathy subgroups. The overall model accuracy and kappa statistics are reported to determine classification effectiveness.

#### Multinomial logistic regression

Two MLR models (i.e., one per intensity/difficulty level) were computed with emotion recognition error rates for each basic emotion (i.e., happiness, surprise, fear, sadness, disgust, and anger) as predictor variables and psychopathy subgroups (CD + CU, CD + GM, CD + II, CD + CU + GM, CD + CU + II, CD + GM + II, and CD + CU + GM + II) as the dependent (i.e., outcome) variable (Hosmer et al., 2013). The CD‐only group was set as the reference group for between‐group comparisons. To isolate the effect of emotion recognition on psychopathy subgroup classification, we employed residualized emotion recognition error rates, adjusting for age, sex, IQ, SES, study site, and the most common co‐occurring psychiatric disorders observed in the sample. This was achieved by regressing out covariates using linear models and extracting residuals for each emotion recognition variable. Odds ratios (ORs) and 95% confidence intervals (CIs) were estimated to assess the impact of each predictor on subgroup membership. To evaluate model assumptions, multicollinearity diagnostics were performed using variance inflation factors. The linearity assumption of log‐odds was tested using Box–Tidwell transformations, ensuring that predictor variables met the proportional odds assumption. We used a two‐tailed *p*‐value threshold of ≤0.05 to indicate statistical significance, without applying corrections for multiple testing due to the exploratory nature of this study.

## RESULTS

### SVM

The supervised SVM model achieved a high overall classification accuracy of 90.5% (*p* < 0.001, 95% CI: 83.2%–95.3%). This confirms that the eight psychopathy subgroups could be reliably (and thus meaningfully) distinguished based on the 75th percentile cut‐off scores of the three YPI subscales (CU, GM, and II traits) selected for subgroup assignment (Table 2). The highest classification accuracy was observed for the CD + CU + II and CD + CU + GM + II subgroups, both of which had a sensitivity of 100% (i.e., the probability of being correctly assigned to this subgroup if one does belong to this subgroup) and a specificity of 100% (i.e., the probability of not being correctly assigned to this subgroup if one does not belong to this subgroup). The lowest classification accuracy was observed for the CD + GM subgroup (75.0% sensitivity, 96.9% specificity). Kappa was very high (0.86), indicating excellent agreement between predicted and actual subgroup membership.

**TABLE 2:** Support vector machine (SVM) classification performance for the eight predefined psychopathy subgroups based on the YPI subscale scores (i.e., 75th percentile cut‐offs).

	CD‐only	CD + CU	CD + GM	CD + II	CD + CU + GM	CD + CU + II	CD + GM + II	CD + CU + GM + II
Sensitivity	0.89	1.0	0.75	0.78	1.0	1.0	1.0	1.0
Specificity	0.96	0.99	0.97	0.98	0.99	1.0	0.99	1.0
Positive predictive value	0.96	0.9	0.67	0.78	0.83	1.0	0.83	1.0
Negative predictive value	0.89	1.0	0.98	0.98	1.0	1.0	1.0	1.0
Balanced accuracy	0.93	0.99	0.86	0.88	1.0	1.0	1.0	1.0

Abbreviations: CD, conduct disorder; CU, callous‐unemotional, GM, grandiose‐manipulative; II, impulsive‐irresponsible; YPI, Youth Psychopathy Inventory (self‐report).

### MLR

**FIGURE 1:**
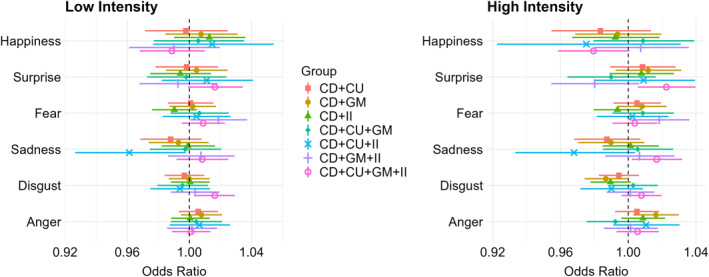
Forest plots illustrate ORs with 95% confidence intervals (adjusted for age, sex, IQ, SES, site, and major co‐occurring psychiatric disorders) from the two separate multinomial regression models per emotion and psychopathy subgroup at the two different intensities in which each emotion was presented—low intensity equals 70%, whereas high intensity equals 90%. ORs greater than 1 indicate a higher error rate and less than 1 indicate a lower error rate compared to the CD‐only subgroup. CD, conduct disorder; IQ, intelligence quotient; ORs, odds ratios; SES, socioeconomic status.

All results for the two MLR models are displayed in Figure 1 and Table 3.

**TABLE 3:** Group comparisons for emotion recognition accuracy per psychopathy subgroup versus CD‐only subgroup.

	CD + CU (*n* = 45)	CD + GM (*n* = 42)	CD + II (*n* = 49)	CD + CU + GM (*n* = 26)	CD + CU + II (*n* = 18)	CD + GM + II (*n* = 27)	CD + CU + GM + II (*n* = 50)
	OR [95% CIs]						
Happiness							
Low	1.00 [0.97, 1.02]	1.01 [0.98, 1.03]	1.01 [0.99, 1.04]	1.01 [0.98, 1.04]	1.01 [0.98, 1.05]	0.99 [0.96, 1.02]	0.99 [0.97, 1.01]
High	0.98 [0.95, 1.01]	0.99 [0.97, 1.02]	0.99 [0.97, 1.02]	1.01 [0.98, 1.04]	0.98 [0.92, 1.03]	1.01 [0.98, 1.04]	0.98 [0.96, 1.00]
Surprise							
Low	1.00 [0.98, 1.02]	1.00 [0.99, 1.02]	0.99 [0.97, 1.01]	1.00 [0.97, 1.02]	0.99 [0.97, 1.02]	0.99 [0.97, 1.02]	1.02 [1.00, 1.03]
High	1.01 [0.99, 1.03]	1.01 [0.99, 1.03]	1.01 [0.99, 1.03]	0.99 [0.96, 1.02]	1.01 [0.98, 1.04]	0.98 [0.95, 1.01]	**1.02 [1.01, 1.04]** ^**b**^
Fear							
Low	1.00 [0.99, 1.02]	1.00 [0.99, 1.02]	0.99 [0.98, 1.00]	1.01 [0.99, 1.03]	1.00 [0.98, 1.03]	**1.02 [1.00, 1.04]** ^**a**^	1.01 [1.00, 1.02]
High	1.01 [0.99, 1.02]	1.01 [0.99, 1.02]	0.99 [0.98, 1.01]	1.01 [0.99, 1.03]	1.00 [0.98, 1.02]	**1.02 [1.00, 1.04]** ^**a**^	1.00 [0.99, 1.02]
Sadness							
Low	0.99 [0.97, 1.01]	0.99 [0.97, 1.01]	1.00 [0.98, 1.02]	1.00 [0.97, 1.02]	**0.96 [0.93, 1.00]** ^**a**^	1.01 [0.99, 1.03]	1.01 [0.99, 1.03]
High	0.99 [0.97, 1.01]	0.99 [0.97, 1.01]	1.00 [0.98, 1.02]	1.01 [0.99, 1.03]	0.97 [0.93, 1.00]	1.01 [0.99, 1.03]	**1.02 [1.00, 1.03]**
Disgust							
Low	1.00 [0.98, 1.01]	1.00 [0.99, 1.01]	1.00 [0.99, 1.01]	1.00 [0.98, 1.01]	0.99 [0.97, 1.01]	1.00 [0.99, 1.02]	**1.02 [1.00, 1.03]** ^**a**^
High	0.99 [0.98, 1.01]	**0.99 [0.97, 1.00]** ^**a**^	0.99 [0.98, 1.00]	1.00 [0.99, 1.02]	0.99 [0.97, 1.01]	1.00 [0.99, 1.02]	1.01 [1.00, 1.02]
Anger							
Low	1.01 [0.99, 1.02]	1.1 [1.0, 1.02]	1.00 [0.99, 1.01]	1.01 [0.99, 1.02]	1.1 [0.99, 1.03]	1.00 [0.99, 1.02]	1.00 [0.99, 1.01]
High	1.01 [0.99, 1.02]	**1.02 [1.00, 1.03]** ^**a**^	1.01 [1.00, 1.02]	0.99 [0.98, 1.01]	1.01 [0.99, 1.03]	1.00 [0.99, 1.02]	1.01 [0.99, 1.02]

*Note*: The table shows the odds ratios (OR) of each psychopathy subgroup compared with the CD‐only subgroup, which was the reference group for between‐group comparisons in the two separate multinomial regression models per intensity level (low: 70% intensity, high: 90% intensity). The ORs and confidence intervals (CIs) are based on the error rates for each of the six basic emotions, with ORs greater than 1 indicating a higher error rate and less than 1 indicating a lower error rate compared to the CD‐only subgroup. Significant findings are in bold with ^a^ *p* < 0.05 and ^b^ *p* < 0.01.

Abbreviations: CD, conduct disorder; CU, callous‐unemotional; GM, grandiose‐manipulative; II, impulsive‐irresponsible.

#### Low intensity (70%) emotions

No significant subgroup differences were observed for happiness, surprise, and anger. However, compared with the CD‐only subgroup, the CD + GM + II subgroup showed *higher* error rates for fear (OR = 1.02 [1.00, 1.04], *p* < 0.05), while the CD + CU + II subgroup showed *lower* error rates for sadness (OR = 0.96 [0.93, 1.00], *p* < 0.05). Additionally, the CD + CU + GM + II subgroup demonstrated *higher* error rates for disgust (OR = 1.02 [1.00, 1.03], *p* < 0.05).

#### High intensity (90%) emotions

There were again no significant subgroup differences for happiness. However, compared with the CD‐only subgroup, the CD + GM subgroup showed *lower* error rates for disgust (OR = 0.99 [0.97, 1.00], *p* < 0.05), but *higher* error rates for anger (OR = 1.02 [1.00, 1.03], *p* < 0.05). Interestingly, the CD + GM + II subgroup again had *higher* error rates for fear (OR = 1.02 [1.00, 1.04], *p* < 0.05). Additionally, the CD + CU + GM + II subgroup showed *higher* error rates for surprise (OR = 1.02 [1.01, 1.04], *p* < 0.01) and for sadness (OR = 1.02 [1.00, 1.03], *p* < 0.05).

Notably, the CD + CU subgroup did not differ significantly from the CD‐only subgroup in terms of emotion recognition accuracy for any of the emotions at either low or high intensity.

## DISCUSSION

The primary aim of this study was to examine emotion recognition accuracy in relation to the three facets of psychopathy in youths with CD (i.e., high levels of CU, GM, and/or II traits). We focused on the six basic emotions, including the distress emotions of fear and sadness, each presented at either low or high intensity (i.e., the facial expressions were harder or easier to recognize, respectively). For this purpose, we created eight mutually exclusive subgroups of CD youths who scored either above or below the 75th percentile cut‐off on the three psychopathy subscales of the YPI, measuring CU, GM, and II traits, respectively. Subgroup sizes varied widely: For example, ∼52% of the entire sample were allocated to the CD‐only subgroup, whereas only ∼3% were allocated to the CD + CU + II subgroup, which was the smallest subgroup of the eight. However, ML analyses strongly supported the validity of these subgroup assignments based on the YPI cut‐off scores. Importantly, differences in sample size did not appear to substantially affect ML classification accuracy, including sensitivity and specificity, which varied within a clinically acceptable range of 75%–100% (Cortese et al., 2023). Interestingly, when focusing on the CD + CU + GM + II subgroup—hypothetically to be at the highest risk of developing adult psychopathy—we found that approximately 9% of youths with CD fell into this category; a proportion consistent with previous studies (Campbell et al., 2004). Importantly, our subgrouping strategy was not intended to imply discrete diagnostic categories but instead reflects empirically distinguishable profiles based on specific combinations of psychopathy traits. This approach aligns with the rationale behind established clinical specifiers (e.g., the LPE specifier in the DSM‐5) and extends previous research by incorporating all three trait dimensions—rather than focusing solely on CU traits. It offers a flexible yet structured framework for modeling complex constellations of psychopathy traits in individuals with CD.

In line with our hypothesis, we found that the CD + CU + GM + II subgroup exhibited the most pervasive impairment in terms of higher error rates for recognizing low intensity disgust as well as high intensity sadness and surprise. However, high CU traits were not necessarily associated with emotion recognition impairments, let alone specifically for distress emotions, such as fear and sadness. Although we did not have strong predictions regarding the effects of intensity/difficulty level on emotion recognition accuracy, no clear pattern of results emerged. Interestingly, the subgroup of CD youths and a combination of high GM and II traits showed particularly consistent impairments in the recognition of fear under both low and high intensity conditions. However, all observed effects were small and must therefore be interpreted with caution until replicated in independent samples.

This is the first study to investigate the extent to which different psychopathy subgroups of CD—considering high CU, GM, and II traits separately and in combination—do or do not exhibit distinct emotion recognition impairments when compared to youths with CD alone. Our data complement and extend the inconsistent and sometimes conflicting findings on the relationship between emotion recognition accuracy, particularly regarding distress emotions, and psychopathy traits in CD (Dawel et al., 2012; Díaz‐Vázquez et al., 2024)—although most previous relevant work has mainly examined high CU traits as a proxy for psychopathy in youths with CD, or high levels of psychopathy independent of its three types of traits (some exceptions are reviewed in Díaz‐Vázquez et al., 2024). Importantly, our findings do not strongly support the influential neurocognitive models that psychopathy (mainly focusing on high CU traits) in youths with CD is closely associated with an impairment in recognizing distress emotions, such as fear and sadness (Blair, 2013); indeed, this is consistent with the conclusions of recent systematic reviews and meta‐analyses (Dawel et al., 2012; Díaz‐Vázquez et al., 2024). However, we found that the subgroup of youths with CD with the most severe manifestation of psychopathy in terms of a combination of high CU, GM, and II traits had problems recognizing sadness and disgust—the latter of which is likely to be a distress emotion as are fear and sadness (Davey, 2011)—as well as the non‐distress emotion of surprise. Interestingly, it was the CD + GM + II subgroup, lacking high CU traits, that demonstrated the most consistent impairment in the recognition of fear but not of other distress emotions. Thus, it is possible that some unique combinations of high levels from the three psychopathy traits, beyond high CU traits alone, contribute to different impairments in recognizing distress emotions. But again, given the novel but exploratory nature of these findings, accompanied by small effect sizes, one must be cautious in drawing any firm conclusions before they are independently verified. However, it would be interesting to determine using longitudinal designs whether youths from these specific psychopathy subgroups have the highest risk of developing psychopathy into adulthood, and what role the impairments of recognizing different distress emotions may play in this regard (Chae & Bolton, 2022).

There are several possible sources that may explain some of the discrepancies regarding the (partial) lack of associations between high CU traits, or psychopathy more broadly, and impairments in recognizing the core distress emotions of fear and sadness, as found in this study and related work (some detailed discussions of this topic can be found in Dawel et al., 2012; Díaz‐Vázquez et al., 2024). For example, the phenotypic context in which high CU traits occur may be important—whether or not they are present in combination with (i) other psychopathy traits—the focus of the current study—, (ii) subclinical manifestations of CD symptoms, and/or (iii) high levels of anxiety. In addition, methodological factors may play a role, such as the composition of the study samples (e.g., CD diagnosis vs. a combination of other disruptive behavior disorders, such as CD and oppositional defiant disorder or even attention deficit hyperactivity disorder [ADHD]), the psychopathy measures (e.g., YPI vs. Antisocial Process Screening Device; Frick & Hare, 2001), the emotion recognition tasks (e.g., verbal labeling vs. nonverbal matching), or the types of emotion stimuli (e.g., static vs. dynamic facial expressions). Clearly, more research is needed to systematically address these potentially influential factors in follow‐up studies.

Our study had several strengths (see also Kohls, Baumann, et al., 2020; Kohls, Fairchild, et al., 2020), including one of the largest representative, mixed‐sex, and well‐characterized samples of youths with CD in this area of research; a clinically motivated stratification approach using 75th percentiles as established and easily reproducible cut‐off scores for subgroup assignment, which is consistent with the idea of psychopathy traits as specifiers for CD diagnosis; and multivariate statistics (e.g., multinomial regression models) that controlled for several potentially confounding variables, such as age, sex, IQ, SES, and site, as well as major co‐occurring psychiatric disorders with the potential to seriously influence results (e.g., ADHD in the context of high II traits; Frick, 2022); a confirmatory ML approach to evaluate whether the clinically motivated, cut‐off‐based subgroups could be reliably distinguished based on the different underlying continuous psychopathy dimensions—this added an important methodological validation step to the subgrouping strategy, and its positive results support its reproducibility and interpretability.

However, our study also had some limitations: First, one must be very cautious about simply adapting the multidimensional construct of adult psychopathy to specify subgroups of youths with CD, as there are still substantial gaps in crucial knowledge on this topic, particularly with respect to the GM and II dimensions (for a critical discussion, see Frick, 2022 and the rebuttal by Salekin, 2022). Second, we used the well‐established and widely used YPI, which, however, only assesses psychopathic traits in the form of self‐report and does not provide any information about the duration of their presence (note that the LPE specifier for CD, according to DSM‐5, requires high CU traits to be present for at least 12 months and in multiple contexts). Third, an “extreme‐groups” design using the 25th percentile (low traits) and 75th percentile (high traits) per YPI subscale as cut‐offs for subgroup stratification would have best served the study aims (Fisher et al., 2020). In fact, this was our initial subgrouping approach, although unfortunately it resulted in some subgroups being unreasonably small to analyze (e.g., *n* < 5). Fourth, although the SVM classifier confirmed that the subgroups could be reliably distinguished based on YPI subscale scores, some of the smaller subgroups may still lack sufficient statistical power to detect subtle effects. Fifth, the cross‐sectional design limits our ability to infer causal mechanisms linking different psychopathy traits to neurocognitive impairments. Finally, while our clinically motivated categorization approach provides theoretical clarity, it may oversimplify the inherently dimensional nature of psychopathy traits.

## CONCLUSION

In conclusion, the results of this large multisite study suggest that it may be more important to consider the presence of different constellation of the three psychopathy traits, rather than just a single trait, to better understand the manifestation of the broad phenotype of CD in terms of potentially contributing neurocognitive mechanisms such as emotion recognition impairments. It appears that no single psychopathy trait, even high CU traits, is necessarily linked to a unique impairment in recognizing basic emotions among those diagnosed with CD, including distress emotions like fear and sadness. Although the effects we observed were independent of potential confounds, they were still quite small, suggesting that there is considerable neurocognitive variability (including overlap in task performance) within each of the eight psychopathy subgroups of youths with CD examined here. Taken together, these findings call into question the central nature of CU traits in the current neurocognitive models of CD, particularly with respect to emotion recognition impairments of distress emotions. However, more research is needed to support such a conclusion. This research should also focus on more complex emotions beyond the basic ones displayed across different modalities (including facial, bodily, and vocal cues) and ideally use more ecologically‐valid stimulus sets, such as video clips that portray the full range of emotional expression experienced by youths in real‐life social situations.

## AUTHOR CONTRIBUTIONS

**Gregor Kohls**: Writing—original draft; conceptualization; funding acquisition; methodology; formal analysis; investigation. **Anka Bernhard**: Writing—review and editing; investigation. **Graeme Fairchild**: Writing—review and editing; funding acquisition. **Christina Stadler**: Writing—review and editing; funding acquisition. **Lucres Jansen**: Writing—review and editing. **Stephane A. De Brito**: Writing—review and editing; funding acquisition. **Veit Roessner**: Writing—review and editing; funding acquisition. **Christine M. Freitag**: Funding acquisition; project administration; writing—review and editing. **Kerstin Konrad**: Writing—review and editing; funding acquisition. **Erik M. Elster**: Writing—review and editing; conceptualization; formal analysis; validation; visualization; methodology; software.

## CONFLICT OF INTEREST STATEMENT

C.M.F. receives royalties for books on Attention‐Deficit/Hyperactivity Disorder, Autism Spectrum Disorder and Major Depressive Disorder. The remaining authors declare no conflicts of interest.

## ETHICAL CONSIDERATIONS

Ethical approval from the relevant local ethics committees for the FemNAT‐CD project was obtained as follows. Aachen: Ethik Kommission Medizinische Fakultät der Rheinisch Westfälischen Technischen Hochschule Aachen (EK027/14). Amsterdam: Medisch Etische Toetsingscommissie (2014.188). Athens: Election Committee of the First Department of Psychiatry, Eginition University Hospital (641/9.11.2015). Barcelona: Child and Adolescent Mental Health—University Hospital Mutua Terrassa (acta 12/13). Basel: Ethik Kommission Nordwest‐und Zentralschweiz (EKNZ 336/13). Bilbao: Hospital del Basurto. Birmingham and Southampton: University Ethics Committee and National Health Service Research Ethics Committee (NRES Committee West Midlands, Edgbaston; REC reference 13/WM/0483). Dublin: SJH/AMNCH Research Ethics Committee (2014/04/Chairman (3)). Frankfurt: Ethik Kommission Medizinische Fakultät Goethe Universität Frankfurt am Main (445/13). Szeged (Hungary): Egészségügyi Tudományos Tanács Humán Reprodukciós Bizottság (CSR/039/00392–3/2014). The study was conducted in accordance with the ethical standards of the Declaration of Helsinki of 1964 and its subsequent amendments. All participants over the legal age of consent (16 years in the UK, otherwise 18 years) in their country gave written informed consent to participate. Participants under the age of consent provided written informed consent, and written informed consent was obtained from parents or legal guardians.

## Supporting information

Supporting Information S1

## Data Availability

The data that support the findings of this study are available from the corresponding author upon reasonable request.
