# Supplementary material for: Psychopathy traits and their link to emotion recognition impairments in conduct disorder
Source: JCPP Adv. 2025 Sep 30;6(2):e70055. doi: 10.1002/jcv2.70055 (PMC13260674; doi:10.1002/jcv2.70055)
Supplement: Supplementary file 1 — Supporting Information S1 [file JCV2-6-e70055-s001.docx]

**Psychopathy traits and their link to emotion recognition impairments in conduct disorder**

**Supporting Information**

**Appendix S1: Supplementary results**

***Correlation between psychopathy dimensions across the entire sample***

The three YPI dimensions (i.e., CU, GM, and II traits) were significantly correlated with each other across the entire of sample of youths with CD. A strong positive association was found between CU and GM traits (*ρ*=.56, *p*<.001), as well as between GM and II traits (*ρ*=.57, *p*<.001). The correlation between CU and II was also significant but slightly weaker (*ρ*=.43, *p*<.001). All correlations are based on Spearman’s rho.

**Table S1. Partial Spearman Correlations**

|  | **CU** | **GM** | **II** |
| --- | --- | --- | --- |
| **Error rates** | *rho* (*p*-value) | | |
| **Happiness:**  High  Low | 0.01 (0.89)  0.02 (0,58) | 0.02 (0.58)  0.05 (0.27) | 0.03 (0.52)  0.02 (0.59) |
| **Surprise:**  High  Low | 0.01 (0.78)  -0.01 (0.83) | 0.06 (0.17)  0.06 (0.19) | 0.01 (0.82)  0.00 (0.91) |
| **Fear:**  High  Low | 0.01 (0.89)  0.00 (0.97) | **0.09 (0.03)**  **0.09 (0.04)** | -0.03 (0.45)  -0.01 (0.79) |
| **Sadness:**  High  Low | -0.07 (0.11)  -0.03 (0.43) | 0.06 (0.18)  0.02 (0.67) | 0.03 (0.43)  0.03 (0.45) |
| **Disgust:**  High  Low | 0.06 (0.20)  0.07 (0.11) | 0.04 (0.41)  0.03 (0.44) | -0.01 (0.77)  0.01 (0.74) |
| **Anger:**  High  Low | 0.03 (0.49)  0.03 (0.50) | 0.03 (0.46)  0.03 (0.45) | 0.05 (0.29)  0.02 (0.66) |
| **Note:** The table shows the partial Spearman correlations (*rho* and *p*-values) between each psychopathy dimension and error rates in emotion recognition for the six basic emotions (low: 70% intensity, high: 90% intensity). Each correlation controls for the other two psychopathy dimensions, isolating the unique association of the dimension of interest. Importantly, the error rate variables were residualized to account for relevant covariates (i.e., age, sex, IQ, SES, site, and comorbidities), ensuring that the reported effects are not confounded. Significant correlations are displayed in **bold** with *p*<.05. CU=callous-unemotional; GM=grandiose-manipulative; II=impulsive-irresponsible. The correlation coefficients can be interpreted as weak *(r* < 0.30), moderate (0.30 < *r* < 0.50), and strong (*r* ≥ 0.50). | | | |

***Exploratory linear regression***

We additionally conducted exploratory linear regression analyses using continuous CU, GM, and II trait scores as predictors of residualized emotion recognition error rates. While these models provide a dimensional perspective, they rely on strong assumptions of linearity and additivity and do not account well for the possibility of non-linear or interactive effects between psychopathy traits. Moreover, given the high intercorrelations among CU, GM, and II traits and the large number of separate models required, this approach increases the risk of type I errors and may yield results that are difficult to interpret in a clinically meaningful way. For these reasons, we consider the categorical subgroup approach our primary analytic strategy. Nevertheless, to ensure full transparency, we report the dimensional regression results below.

For happy faces (70% and 90% intensity), none of the predictors reached significance with both models being not significant (Happy70: *F*(3,534)=1.25, *p*=.29; Happy90: *F*(3,534)=1.30, *p*=.27)

For surprise recognition, results were likewise not significant (Surprise70: *F*(3,534)=1.43, *p*=.23; Surprise90: *F*(3,534)=2.27, *p*=.08).

For fear recognition, both models were significant (Fear70: *F*(3,534)=3.06, *p*=.028,*R²*=.017; Fear90: *F*(3,534)=2.75, *p*=.042, *R²*=.015). In both, higher GM scores significantly predicted greater error rates (Fear70: *b*=0.30, *SE*=0.13, *t*=2.39, *p*=.01; Fear90: *b*=0.33, *SE*=0.13, *t*=2.48, *p*=.013). CU and II traits were not significant (all *ps*>0.076).

For sad faces, models were not significant at both intensities (Sad70: *F*(3,534)=0.77, *p*=.51; Sad90: *F*(3,534)=1.60, *p*=.19).

For disgust recognition, models were nonsignificant at both intensities (Disgust70: *F*(3,534)=2.20, *p*=.088; Disgust90: *F*(3,534)=1.03, *p*=.38).

For anger recognition, neither model was significant (Anger70: *F*(3,534)=1.28, *p*=.28; Anger90: *F*(3,534)=1.20, *p*=.31).

**Table S2. SVM Prediction Results**

|  | **Reference** | |  |  |  |  |  |  |
| --- | --- | --- | --- | --- | --- | --- | --- | --- |
| **Prediction** | **CD**  **only** | **CD+**  **CU** | **CD+**  **GM** | **CD+**  **II** | **CD+**  **CU+GM** | **CD+**  **CU+II** | **CD+**  **GM+II** | **CD+**  **CU+GM+**  **II** |
| CD only | 50 | 0 | 0 | 2 | 0 | 0 | 0 | 0 |
| CD+CU | 1 | 9 | 0 | 0 | 0 | 0 | 0 | 0 |
| CD+GM | 3 | 0 | 6 | 0 | 0 | 0 | 0 | 0 |
| CD+II | 2 | 0 | 0 | 7 | 0 | 0 | 0 | 0 |
| CD+CU+GM | 0 | 0 | 1 | 0 | 5 | 0 | 0 | 0 |
| CD+CU+II | 0 | 0 | 1 | 0 | 0 | 3 | 0 | 0 |
| CD+GM+II | 0 | 0 | 0 | 0 | 0 | 0 | 5 | 0 |
| CD+CU+GM+II | 0 | 0 | 0 | 0 | 0 | 0 | 0 | 10 |
| **Note:** CD=conduct disorder; CU=callous-unemotional; GM=grandiose-manipulative; II=impulsive-irresponsible. | | | | | | | | |
